# RETRACTION: Trace elements-based Auroshell gold@hematite nanostructure: Green synthesis and their hyperthermia therapy

**DOI:** 10.1049/nbt2/9878750

**Published:** 2025-02-28

**Authors:** IET Nanobiotechnology

RETRACTION: H. M. Alahdal, S. A. Abdullrezzaq, H. I. M. Amin, S. F. Alanazi, A. T. Jalil, M. Khatami, and M. M. Saleh, “Trace elements-based Auroshell gold@hematite nanostructure: Green synthesis and their hyperthermia therapy,” *IET Nanobiotechnology* 17, no. 1 (2023): 22–31, https://doi.org/10.1049/nbt2.12107.

The above article, published online on 24 November 2022 in Wiley Online Library (wileyonlinelibrary.com), has been retracted by agreement between the journal Editor-in-Chief; The Institution of Engineering and Technology; and John Wiley & Sons Ltd.

The retraction has been agreed due to concerns raised by a third party. An investigation revealed several inconsistencies regarding the experiments described and the results presented. The experimental methods are not described in detail, and so the research described is not fully comprehensible for readers, not reproducible, and the editors consider the conclusions invalid. The authors were contacted for an explanation but have not addressed any of the concerns. They have been informed of the decision to retract and they disagree with it.

